# Supplementary material for: A new scoring system with simple preoperative parameters as predictors of early recurrence of pancreatic ductal adenocarcinoma
Source: PLoS One. 2023 Jul 14;18(7):e0288033. doi: 10.1371/journal.pone.0288033 (PMC10348551; doi:10.1371/journal.pone.0288033)

**Supplementary Information**

**A new scoring system with simple preoperative parameters as predictors of early recurrence of pancreatic ductal adenocarcinoma**

Tomonari Shimagaki^1^, Keishi Sugimachi^1^, Yohei Mano^1^, Takahiro Tomino^1^, Emi Onishi^1^, Yuichiro Nakashima^2^, Masahiko Sugiyama^2^, Manabu Yamamoto^2^, Masaru Morita^2^, Mototsugu Shimokawa^3^, Tomoharu Yoshizumi^4^, Yasushi Toh^2^

^1^Department of Hepatobiliary and Pancreatic Surgery, National Hospital Organization Kyushu Cancer Center, Fukuoka 811-1395, Japan

^2^Department of Gastroenterological Surgery, National Hospital Organization Kyushu Cancer Center, Fukuoka 811-1395, Japan

^3^Department of Biostatistics, Yamaguchi University Graduate School of Medicine, Yamaguchi, 755-8505, Japan

^4^Department of Surgery and Science, Graduate School of Medical Sciences, Kyushu University, 3-1-1 Maidashi, Fukuoka 812-8582, Japan

**Supplementary Table 1.** Comparison of tumor size and pathological findings

| Variable | Tumor size ≧3.1cm  (n=60) | Tumor size < 3.1cm (n=93) | *P value* |
| --- | --- | --- | --- |
| Lymph node metastasis (yes/no) | 45/15 | 49/44 | **0.0056** |
| Macroscopic classification;  nodular /infiltrative/  cystic/mixed type | 7/51/1/1 | 17/78/0/0 | 0.3067 |
| ly (0/1) | 46/14 | 84/9 | **0.0210** |
| v (0/1) | 32/28 | 71/22 | **0.0030** |
| ne (0/1) | 8/52 | 22/71 | 0.1164 |
| CH (0/1) | 48/12 | 75/18 | 0.9218 |
| DU (0/1) | 38/22 | 80/13 | **0.0011** |
| S (0/1) | 15/45 | 49/44 | **0.0007** |
| RP (0/1) | 8/52 | 39/54 | **0.0002** |
| PV (0/1) | 43/17 | 81/12 | **0.0174** |
| A (0/1) | 57/3 | 90/3 | 0.5810 |
| PL (0/1) | 54/6 | 88/5 | 0.2797 |
| OO (0/1) | 57/3 | 90/2 | 0.3397 |

ly, invasion to lymphatic vessels; 0/1, no/yes; v, invasion to veins; ne, invasion to nerves; CH, distal bile duct invasion; DU, duodenal invasion; S, serosal invasion; RP, retropancreatic tissue invasion; PV, portal venous system invasion; A, arterial system invasion; PL, extrapancreatic nerve plexus invasion; OO, invasion of other organs.

**Supplementary Figure Legends**

**Supplementary Figure 1.** Kaplan–Meier analysis of overall survival of patients who underwent pancreatectomy for pancreatic ductal adenocarcinoma stratified by the presence or absence of (A) preoperative treatment and (B) postoperative adjuvant chemotherapy.


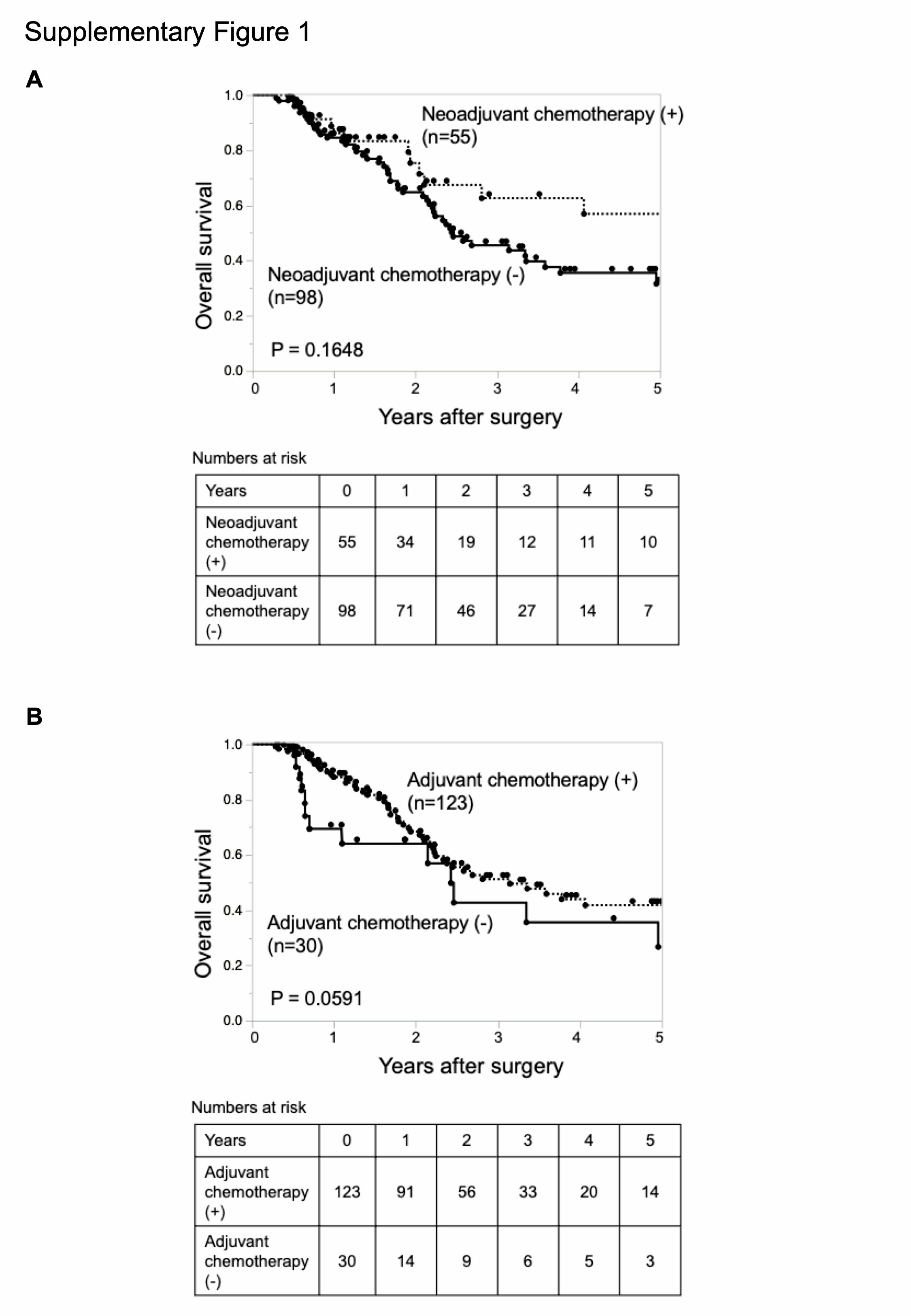


**Supplementary Figure 2.** Kaplan–Meier analysis of (A, B) disease-free survival and (C, D) overall survival of patients who underwent pancreatectomy for pancreatic ductal adenocarcinoma stratified by the prediction scoring system with three factors by combining the parameters before NAT with 55 NAT cases and the pre-operative parameters without 98 NAT cases.

NAT, neoadjuvant therapy.


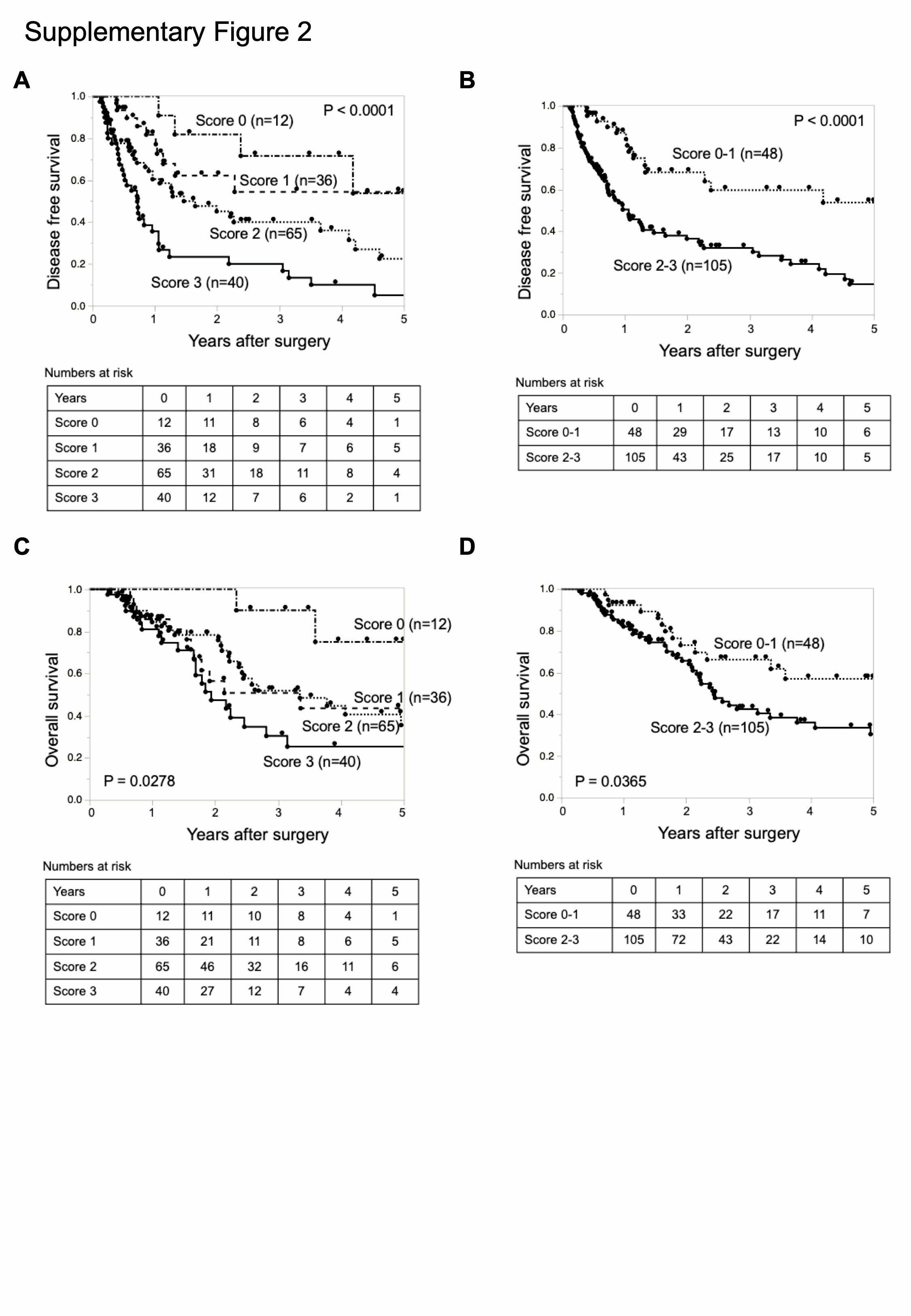


**Supplementary Figure 3.** In 55 cases that underwent NAT, Kaplan–Meier analysis of (A, B) overall survival and (C, D) disease-free survival of patients who underwent pancreatectomy for pancreatic ductal adenocarcinoma stratified by the prediction scoring system using parameters before and after NAT.

NAT, neoadjuvant therapy.


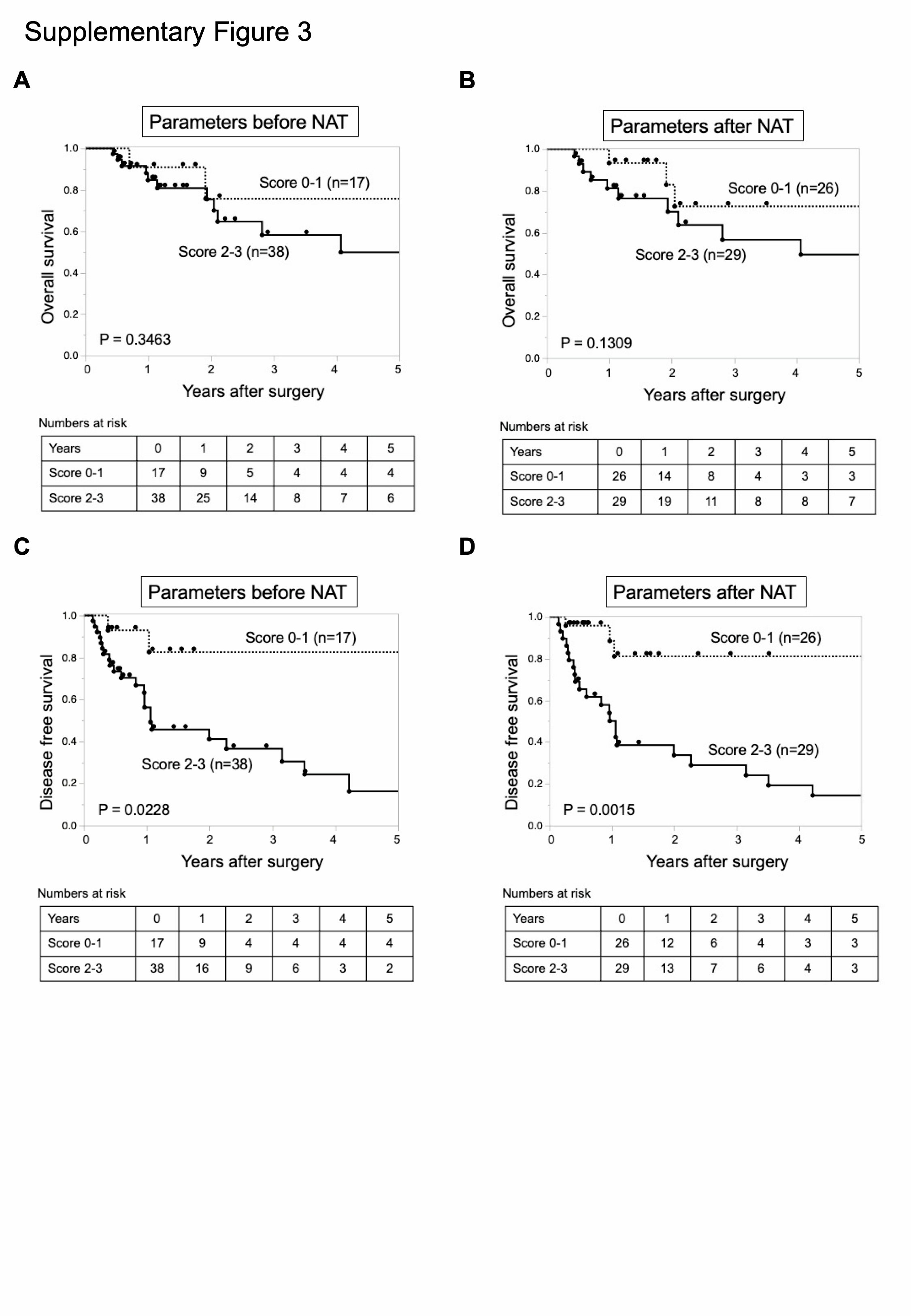

Supplement: S1 File — (DOCX) [file pone.0288033.s001.docx]
