# Supplementary material for: A new scoring system with simple preoperative parameters as predictors of early recurrence of pancreatic ductal adenocarcinoma
Source: PLoS One. 2023 Jul 14;18(7):e0288033. doi: 10.1371/journal.pone.0288033 (PMC10348551; doi:10.1371/journal.pone.0288033)
Supplement: Attachment — Submitted filename: Response to Referees.docx [file pone.0288033.s002.docx]

**Answers to the Reviewer #1:**

We appreciate for the thoughtful comments and suggestions. Our responses to them are as follows. We changed the manuscript accordingly.

**Q1.**

The title of Table 3 was “univariate and multivariate Cox regression analysis for ER”. Is this analysis really Cox regression analysis? Please check by the statisticians. If possible, please add the statisticians as one of the co-authors.

**Our response to Q1:**

We appreciated the reviewer’s comment and Table 3 has been modified for univariate and multivariate analysis using logistic regression analyses. We asked a statistician to review this study and included him as a co-author.

The above is described in the materials and methods section (page 10, lines 13) and in Table 3.

**Q2.**

In the Table 3, only four consecutive parameters were divided into two groups using the ROC analysis. I think that it will not be appropriate to divide only four consecutive parameters, and it will be preferable to divide all consecutive parameters into two groups using the ROC analysis. I recommend that these analysis will be supervised by the statisticians.

**Our response to Q2:**

As suggested by the reviewer, under the guidance of a statistician, all continuous parameters in Table 3 were divided into two groups using ROC analysis, followed by univariate and multivariate analysis.

The above is described in the results section (page 13, lines 5) and in Table 3.

**Q3.**

Authors should show the exact ER rates in every scores. I think that the ER rates of the patients with score 2 or 3, which are majority of this subjects (102 patients/153 patients), are probably similar ER rate of the entire ER rate (35%). So, this score will not actually be useful in majority of the pancreatic cancer patients.

**Our response to Q3:**

Using this new scoring system, the ER rate was calculated to be 0% (0 /16 patients) in the 0-point group, 16.2% (7 /36 patients) in the 1-point group, 40.7% (24 /59 patients) in the 2-point group, and 65.7% (23 /35 patients) in the 3-point group. The higher the score was, the higher the ER rate was.

The above is described in the results section (page 15, lines 1).

**Q4.**

The stratum-specific likelifood ratio (SSLR) indicates by how much a given diagnostic test result will increase or decrease the pretest probability of the target disorder. LR greater than 10 or less than 0.1 generates large and conclusive changes from pretest to post-test probability. Thus SSLR is estimated according to the following formula: SSLR = (x1/n1)/(x0/n0), where x1 is the number of patients in the stratum with ER; n1 is the total number of patients with ER; x0 is the number of patients in the stratum without ER; n0 is the total number of patients without ER (ref; Teasdale G, Jennett B (1974) Assessment of coma and impaired consciousness: a practical scale. Lancet 2:81–84). I recommend the authors to estimate SSLR for each score, and please prove the actual utility of this score. If the SSLR was greater than 10 or less than 0.1, this score will have the conclusive utility.

**Our response to Q4:**

The stratum specific likelihood ratio (SSLR) is the probability of a given test result when the disease is present, divided by the probability of the same test result when the disease is absent. We determined these ratios by means of the formula SSLR = (x1/n1)/(x0/n0), where x1 is the number of patients in the stratum with ER; n1 is the total number of patients with ER; x0 is the number of patients in the stratum without ER; n0 is the total number of patients without ER.

SSLR was calculated to be 0 in the 0-point group and 0.356 in the 1-point group, 1.257 in the 2-point group, and 3.513 in the 3-point group.

The above is described in the materials and methos section (page 10, lines 14) and the results section (page 15, lines 4).

**Q5.**

 I recommend the authors to develop a score using each standardized variable based on the regression coefficient of the logistic regression model, because the odds ratio of each parameters of the score was greatly different. Considering their odds ratio, the equation for the scoring system had better be calculated on the assumption that a patient receives about 3-4 points for high PLR, if a patient receives 1 point for each of high CA19-9 and the tumor diameter of >3.1 cm."

**Our response to Q5:**

We appreciate the reviewer’s comment, and added logistic regression model in the revised manuscript.

We calculated a predictive model for ER after the resection for PDAC by stepwise multiple logistic regression analysis as follows. If a patient had a high PLR, a high CA19-9, or a tumor diameter > 3.1 cm, they received 1 point each in this model.

Logit(*p*) = 1.6 + 1.2 × high PLR + 0.7 × high CA19-9 + 0.5 × tumor diameter > 3.1cm

The AUC, sensitivity, and specificity of our prediction model were 0.763, 85.2% and 55.6%, respectively (Figure 4, Table 2). This ER prediction model was comparable to the scoring system and was more useful than any test for identifying ER after the resection of PDAC (Table 2).

The above is described in the abstract and the results section (page 15, lines 10).

**Answers to the Reviewer #2:**

We appreciate for your thoughtful comments and suggestions. Our responses to the comments are as follows.

**Q.**
The cohort used consisted of NAT (+) and NAT (-) cases. The two groups may differ clinico-pathologically and biologically (e.g., the same T factor but the cancer cell density is sometimes very different between NAT (+) and NAT (-) PDAC). If so, scoring system optimal to predict early recurrence of PDAC may be changed dependent on composition of PDAC with and without NAT in the cohort used. What do the authors think about this?

**Our response to Q:**

We performed this scoring system analysis in total 153 cases combining 55 NAT cases and 98 upfront surgery cases, using parameters measured before NAT (at time of first diagnosis). As a result, the significant worse prognosis for both OS and DFS were observed in the cases with higher score (Supplementary Figure 2A-2D), which was similar to the main results (Figure 3A-3D).

We also did this scoring system analysis only in 55 cases that underwent NAT. We compared OS and DFS stratified by the prediction scoring system using parameters before and after NAT (Supplementary Figure 3A-3D). As a result, both OS and DFS tended to be worse in the cases with higher score, whether the parameters before or after NAT were used. There was no statistically significant difference due to small number of cases. These results indicated that the preoperative parameters have the same significance even if it is measured before or after preoperative chemotherapy.

The above is described in the results section (page 14, lines 9).
