# Supplementary material for: A new scoring system with simple preoperative parameters as predictors of early recurrence of pancreatic ductal adenocarcinoma
Source: PLoS One. 2023 Jul 14;18(7):e0288033. doi: 10.1371/journal.pone.0288033 (PMC10348551; doi:10.1371/journal.pone.0288033)
Supplement: Attachment — Submitted filename: Response to Referees.docx [file pone.0288033.s003.docx]

**Answers to the Reviewer #1:**

We appreciate for the thoughtful comments and suggestions. Our responses to them are as follows. We changed the manuscript accordingly.

**Q1.**

The authors have clearly demonstrated the SSLR in each score. Considering the SSLR in each score, this score will be particularly useful for patients with a score of 0 (SSLR = 0), which accounts for only 16 out of the total 153 patients. However, for the majority of patients (137 out of 153), calculating this score did not significantly alter the probability of early recurrence when compared to the overall early recurrence rate (35%). The authors should mention this limitation of the study in the limitations paragraph within the Discussion section.

**Our response to Q1:**

We appreciated the reviewer’s comment.

We have clearly demonstrated the SSLR in each score. Considering the SSLR in each score, this score will be particularly useful for patients with a score of 0 (SSLR = 0), which accounts for only 16 out of the total 153 patients. However, for the majority of patients (137 out of 153), calculating this score did not significantly alter the probability of ER when compared to the overall ER rate (35%).

The above is described in the discussion section (page 19, lines 14).
